# The efficacy of aspirin versus low‐molecular‐weight heparin for venous thromboembolism prophylaxis after knee and hip arthroplasty: A systematic review and meta‐analysis of randomized controlled trials

**DOI:** 10.1002/ksa.12456

**Published:** 2024-09-03

**Authors:** Loay A. Salman, Seif B. Altahtamouni, Harman Khatkar, Abdallah Al‐Ani, Shamsi Hameed, Abtin Alvand

**Affiliations:** ^1^ Department of Orthopaedic Surgery Surgical Specialty Center, Hamad General Hospital, Hamad Medical Corporation Doha Qatar; ^2^ Royal London Hospital London UK; ^3^ Office of Scientific Affairs and Research King Hussein Cancer Center Amman Jordan; ^4^ Nuffield Orthopaedic Centre University of Oxford Oxford UK

**Keywords:** arthroplasty, DVT, hip, knee, PE, venous thromboembolism

## Abstract

**Purpose:**

The purpose of this study was to assess the efficacy of aspirin versus low‐molecular‐weight heparin (LMWH) in preventing venous thromboembolism (VTE) following hip and knee arthroplasty.

**Methods:**

PubMed/Medline, Embase, Cochrane Library and Google Scholar databases were searched from inception till June 2024 for original trials investigating the outcomes of aspirin versus LMWH in hip and knee arthroplasty. The primary outcome was VTE. Secondary outcomes included minor and major bleeding events, and postoperative mortality within 90 days. This review was conducted per the Preferred Reporting Items for Systematic Reviews and Meta‐Analyses guidelines.

**Results:**

A total of 7 randomized controlled trials with 12,134 participants were included. The mean ages for the aspirin and LMWH cohorts were 66.6 (57.6–69.0) years and 66.8 (57.9–68.9) years, respectively. There was no statistically significant difference in the overall risk of VTE between the aspirin and the LMWH cohorts (odds ratio [OR]: 0.95; 95% confidence interval [CI]: 0.48–1.89; *p*: 0.877). A subanalysis based on the specific VTE entity (pulmonary embolism [PE] or deep venous thrombosis) showed a significantly higher PE risk for patients receiving aspirin than the LMWH cohort (OR: 1.79; 95% CI: 1.11–2.89; *p*: 0.017). There was no difference in minor (OR: 0.64; 95% CI: 0.40–1.04; *p*: 0.072) and major bleeding (OR: 0.77; 95% CI: 0.40–1.47; *p*: 0.424) episodes across both groups. Furthermore, subanalysis among the total knee arthroplasty group showed that the aspirin cohort was significantly more likely to suffer VTEs than their LMWH counterparts (OR: 1.55; 95% CI: 1.21–1.98; *p* < 0.001).

**Conclusion:**

This study demonstrated a significantly higher risk of PE among patients receiving aspirin compared to LMWH following hip or knee arthroplasty for osteoarthritis. Aspirin was associated with a significantly higher overall VTE risk among patients undergoing knee arthroplasty, in particular. This might suggest the inferiority of aspirin compared to LMWH in preventing VTE following such procedures.

**Level of Evidence:**

Level I.

**Abbreviations**
DVT: deep venous thrombosis
LMWH: low‐molecular‐weight heparin
OCEBM: Oxford Centre for Evidence‐Based Medicine
PE: pulmonary embolism
PRISMA: Preferred Reporting Items for Systematic Reviews and Meta‐Analyses
PROSPERO: International Prospective Register of Systematic Reviews
THA: total hip arthroplasty
TKA: total knee arthroplasty
VTE: venous thromboembolism

## INTRODUCTION

Hip and knee arthroplasty are among the most commonly performed orthopaedic procedures worldwide, with over 2.24 million primary and revision surgeries performed annually in the United States alone [35]. By 2030, the annual number of total knee arthroplasty (TKA) and total hip arthroplasty (THA) cases is projected to exceed 1.26 million and 500,000, respectively [2, 36, 40].

Despite their high success rates, venous thromboembolism (VTE) remains a feared complication of knee and hip arthroplasty. Approximately 2% of TKA and THA cases develop symptomatic VTE, and this incidence is expected to rise due to the ageing population and the increasing number of joint replacements globally [3, 4].

Various VTE prophylaxis strategies have been implemented, incorporating mechanical and pharmacological prophylactic measures. However, clinical guidelines have yet to reach a consensus, with varying recommendations [10, 17, 18, 27]. Over the past decade, aspirin has gained popularity as a primary thromboprophylaxis agent following lower limb arthroplasty, supported by several studies highlighting its affordability, safety and ease of administration [1, 25, 42].

Numerous previous studies have compared the efficacy of aspirin versus low‐molecular‐weight heparin (LMWH), including enoxaparin and dalteparin, as commonly utilized postoperative thromboprophylaxis agents in lower limb arthroplasty [4, 19]. However, these studies were often statistically underpowered or had confounding effects due to other utilized agents [4, 19]. A level II systematic review by Farey et al. [11] compared aspirin and enoxaparin (LMWH) for VTE prevention in elective lower limb arthroplasty, involving 4 trials and 1507 patients. It suggested aspirin might be a safe alternative to enoxaparin for TKA within a multimodal enhanced recovery protocol but found no similar benefit for THA. Conversely, the more recent CRISTAL trial [38], with 9711 patients, showed aspirin to be less effective than enoxaparin, with higher VTE rates (3.45% vs. 1.82%; estimated difference, 1.97%; 95% confidence interval [CI], 0.54%–3.41%). In addition to the CRISTAL trial, two more trials were not included in Farey et al.'s review, necessitating a high‐quality, robust, up‐to‐date review on this controversial topic.

Therefore, this systematic review aims to provide an updated analysis of the highest‐quality evidence comparing the efficacy of aspirin versus LMWH in preventing VTE in the immediate postoperative period following hip and knee arthroplasty. Also, a subgroup analysis will be performed to compare both agents in TKA and THA, separately. We hypothesize that aspirin is noninferior to LMWH with a similar overall incidence rate of VTE following hip and knee arthroplasty.

## MATERIALS AND METHODS

This systematic review was conducted according to the Preferred Reporting Items for Systematic Reviews and Meta‐Analyses (PRISMA) guidelines [26]. The protocol was pre‐registered on the International Prospective Register of Systematic Reviews (PROSPERO).

### Search strategy

Four online databases (PubMed/Medline, Embase, Cochrane Library, Google Scholar) were searched from inception to 15 June 2024 to identify all the studies investigating the outcomes of aspirin versus LMWH following TKR and THR. The following keywords and their derivative were included ‘Arthroplasty’ OR ‘replacement’ AND ‘Hip’ OR ‘knee’ AND ‘venous thromboembolism’ OR ‘VTE’ OR ‘Deep Venous thrombosis’ OR ‘DVT’ OR ‘Pulmonary embolism’ OR ‘PE’ AND ‘Aspirin’ AND ‘LMWH’ OR ‘Enoxaparin’ OR ‘Dalteparin’ OR ‘Anticoagulant’. See Supporting Information S1: Appendix for detailed search strategies.

### Eligibility criteria

Studies were considered eligible if they satisfied the following criteria: (1) original RCTs; (2) comparing the outcomes of aspirin to LMWH (enoxaparin or dalteparin); (3) in both knee and hip arthroplasty performed for the treatment of osteoarthritis; (4) no restriction on the type of implants; (5) published in the English language. Exclusion criteria included (1) studies not reporting any of the outcomes of interest (VTE, bleeding, mortality rate); (2) studies comparing combined thromboprophylaxis agents protocol where the standalone effect of aspirin versus LWMH is confounded; (3) observational studies, review articles, preclinical, cadaveric and anatomical studies, and case reports; (4) studies with incomplete or unextractable data for analysis. See Supporting Information S1: Appendix for a detailed PICO framework.

The main outcome of interest was the occurrence of symptomatic or asymptomatic VTE, in the form of pulmonary embolism (PE), deep venous thrombosis (DVT) or both. Secondary outcomes included major bleeding events (organ, limb or muscle compromise requiring urgent saving surgery), minor bleeding events (wound ooze or minor bleed) and mortality within 90 days postoperatively.

### Study screening

Two authors conducted the screening process independently and blindly by screening the titles and abstracts of the retrieved articles. For studies meeting the prespecified eligibility criteria, a full‐text review was performed. Any disagreement between the two authors was resolved by discussion with a more senior author. References of included articles were manually sought to ensure all relevant studies were included.

### Data extraction

Two authors independently and blindly extracted data from the included articles. The following data were collected: studies' characteristics, patients' demographics (such as age, gender and body mass index [BMI]), number of patients, number of knees, number of hips, comorbidities (including previous DVT history, cardiac diseases, diabetes and hypertension), preoperative blood haemoglobin and platelet values, smoking and alcohol status, overall complications, VTE, PE, DVT, major and minor bleeding events, mean follow‐up period and mortality within 90 days.

### Quality assessment

The risk of bias assessment in each study was conducted by two authors independently utilizing the Cochrane risk of bias tool [9]. The included studies were categorized as having high, low or unclear risk of bias based on seven domains: random sequence generation, allocation concealment, selective reporting, blinding of participants and personnel, blinding of outcome assessment, incomplete outcome data and other sources of bias. JADAD score was also utilized to further assess the internal validity and overall quality of included trials [16]. Additionally, an Oxford Centre for Evidence‐Based Medicine (OCEBM) [30] level of evidence (LoE) was determined for each study, followed by an overall GRADE recommendation for the entire review [14], in accordance with the Cochrane Collaboration Handbook.

### Quantitative assessment

The quantitative synthesis of data was conducted using R Statistical Software (version 4.0.2; R Core Team, 2020) using the following packages and functions: ‘meta’, ‘metaprop’, ‘metamean’, ‘metagen’, ‘meta.reg’ and ‘forest’. The study outcomes were either expressed as pooled prevalence or raw means, both with their associated 95% confidence intervals. Heterogeneity among effect sizes was evaluated using *I* ^2^ statistic. Definitions for heterogeneity were adapted from the Cochrane Handbook (>25% mild, 25%–50% moderate, >50% severe). A *p*‐value less than 0.05 was considered significant. Due to the high *I* ^2^ value (i.e., greater than 50%), a random effects model was utilized.

## RESULTS

### Search results

**Figure 1:**
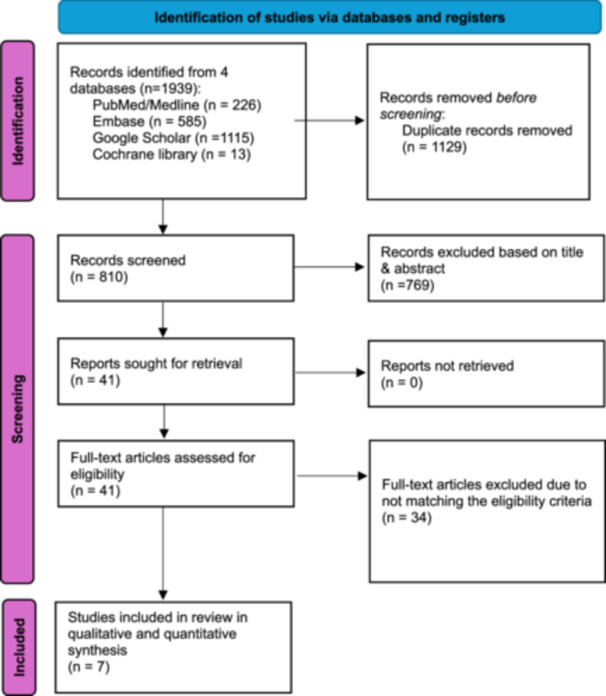
PRISMA flow diagram of record identification, screening and selection in meta‐analysis. PRISMA, Preferred Reporting Items for Systematic Reviews and Meta‐Analyses.

Rayyan website was used to manage the literature search results [31]. The initial database search returned 1939 articles, from which 1129 duplicates were eliminated, leaving 810 records for screening based on title and abstract. Among these, 769 were excluded, leaving 41 papers for full‐text evaluation. Ultimately, seven studies [5, 12, 21, 38, 41, 43, 44] met the eligibility criteria and underwent qualitative and quantitative synthesis. The depiction of this process can be found in Figure 1 of the PRISMA flowchart.

### Quality assessment (LoE and risk of bias)

**Figure 2:**
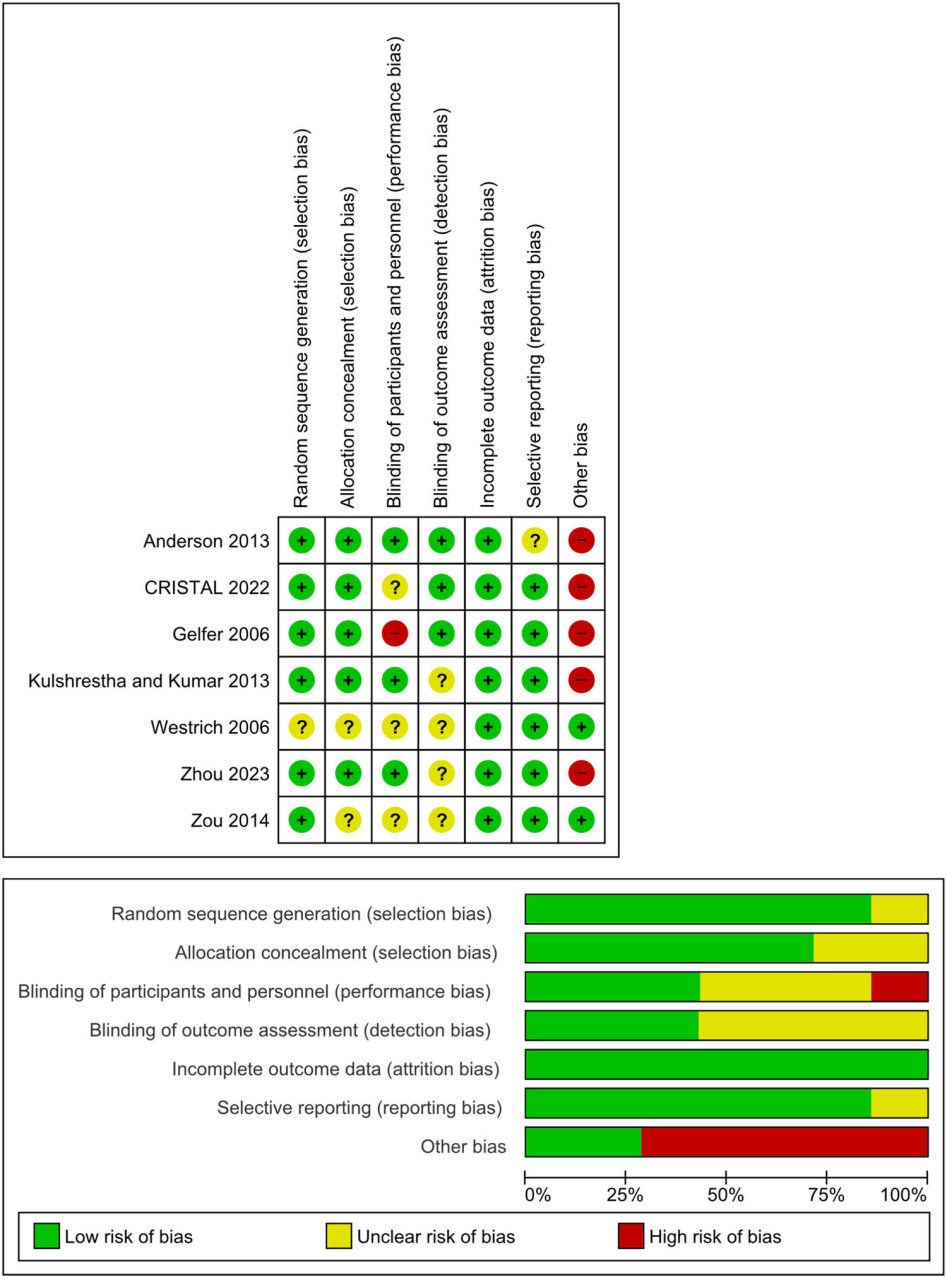
A summary of the quality assessment, according to the Cochrane risk of bias tool. +, low risk; ?, uncertain risk; −, high risk.

Based on the OCEBM criteria, 7 studies were level 2, with an overall GRADE B of recommendation assigned to the review. This grading reflects the coherence of results among the studies included and suggests that this evidence is suitable for recommendation and general adherence in clinical practice [14]. A summary of the quality assessment, according to the Cochrane risk of bias tool, is presented in Figure 2. The JADAD scores of the included trials ranged from 3 to 5 (Supporting Information S1: Appendix), reflecting their high quality. Interpretations of the quality of evidence and proposed recommendations are based on the GRADE handbook and were completed using the GRADEpro tool, which is summarized in Table 1.

**Table 1:** Quality of evidence and proposed recommendations using the GRADEpro tool.

Outcomes	Number of participants	Number of studies	Quality of evidence	OR (95% CI)	Recommendation
Risk of VTE	11,608	7	⊕◯◯◯^a^ ^,^ ^b^ ^,^ ^c^	0.95 (0.48–1.89)	Weak
Risk of DVT	11,608	7	⊕⊕◯◯^a^ ^,^ ^b^	0.99 (0.51–1.92)	Weak
Risk of PE	10,366	4	⊕⊕⊕◯^b^	1.79 (1.11–2.89)	Strong
Risk of major bleeding	11,152	4	⊕⊕⊕◯^b^ ^,^ ^c^	0.77 (0.40–1.47)	Moderate
Risk of minor bleeding	2148	5	⊕⊕⊕◯^b^	0.64 (0.40–1.04)	Strong
Risk of mortality	9981	2	Cannot be estimated	1.03 (0.23–4.62)	N/A

Abbreviations: CI, confidence interval; DVT, deep venous thrombosis; OR, odds ratio; PE, pulmonary embolism; VTE, venous thromboembolism.

^a^ Due to inconsistency of results.

^b^ Due to imprecision.

^c^ Due to publication bias.

### Characteristics of included studies

A total of seven trials were examined in this meta‐analysis (Tables 2 and 3). The total number of observations across the studies was 12,134 comprised of 54.6% in the aspirin cohort and 45.4% in the LMWH cohort. The weighted mean ages for the aspirin and LMWH cohorts were 66.6 (57.6–69.0) and 66.8 (57.9–68.9) years, respectively. The weighted mean BMI values for the aspirin and LMWH cohorts were 30.5 (25.6–30.75) and 30.3 (24.9–30.75) kg/m^2^, respectively. Per available data, females outnumbered males in all studies but one (Anderson) for both aspirin and LMWH cohorts. The female‐to‐male ratios were 1.27 and 1.33 for the aspirin and LMWH cohorts, respectively. In terms of chronic diseases, hypertension was the most commonly reported comorbidity across both the aspirin and LMWH cohorts. The weighted mean haemoglobin values for the aspirin and LMWH cohorts were 138.9 (129.9–141.7) and 138.7 (128.7–141.3), respectively. Mean PT for both cohorts was recorded at 10.1. The meta‐analysis of all outcomes is summarized in Table 4.

**Table 2:** Characteristics of included studies.

Study	Design	Country	Age (Mean ± SD)		Gender (M/F)	BMI (Mean ± SD)		Surgical Procedure (TKA, THA)
			Aspirin	LMWH		Aspirin	LMWH	
Gelfer et al. [12]	RCT	Israel	68 ± 10.4	67 ± 8.7	44/77	28 ± 4	29 ± 4.8	48, 73
Westrich et al. [41]	RCT	United States	69 ± 12.1	68.9 ± 9.6	99/176	NA	NA	264, 0
Kulshrestha and Kumar [21]	RCT	India	NA	NA	328/572	NA	NA	900, 900
Zou et al. [44]	RCT	China	62.7 (Range: 47–79)	65.7 (Range: 54–80)	48/174	27.8 (Range: 17.8–40)	27 (Range: 20.3–37)	222, 222
CRISTAL [38]	RCT	Australia	Median (IQR): 67 (61–74)	Median (IQR): 68 (61–74)	4200/5511	Median (IQR): 30.5 (26.9–35.1)	Median (IQR): 30.6 (26.9–34.9)	9203, 5705
Anderson et al. [5]	RCT	Canada	57.6 ± 11.9	57.9 ± 12.2	444/341	29.3 ± 5.9	27.9 ± 5.8	778, 0
Zhou et al. [43]	RCT	China	66.4 ± 7.6	64.1 ± 6.7	55/65	25.6 ± 3.3	24.9 ± 3.8	120, 120

Study	Mechanical prophylaxis	Enhanced recovery protocol (Y/N)	Diagnostic criteria			Outcomes	
			VTE	Symptomatic DVT	Asymptomatic DVT	Primary	Secondary
Gelfer et al. [12]	Continuous enhanced circulation therapy (CECT), aspirin group only	N	Clinical only	Bilateral ascending venogram 5–8 days postoperatively	Venous duplex ultrasound	asymptomatic and symptomatic DVT	Blood loss, hospitalization days, major and minor bleeding events
Westrich et al. [41]	VenaFlow calf compression device	N	Computed tomogram	Venous duplex ultrasound days 3–5 postoperatively, 4–6 week follow‐up	NA	Asymptomatic DVT	Blood loss, blood transfusion, major and minor bleeding events
Kulshrestha and Kumar [21]	Intermittent pneumatic compression	N	Computed tomogram	Not measured	Venous duplex ultrasound	Symptomatic DVT	Major and minor bleeding events, infection
Zou et al. [44]	NA	Y	NA	Venous duplex ultrasound at 2 and 4 weeks postoperatively	NA	Asymptomatic DVT	Major and minor bleeding events, infection
CRISTAL (2022)	Intermittent pneumatic compression	N	NA	NA	NA	Symptomatic VTE	Joint‐related readmission, joint‐related reoperation, major bleeding events, mortality within 90 days, and adherence rates
Anderson et al. [5]	NA	N	Clinical only	NA	Clinical only	Symptomatic VTE	Death, major bleeding, clinically important nonmajor bleeding, myocardial infarction, stroke and wound infection
Zhou et al. [43]	Compression bandaging and ice packs	N	Computed tomogram	Arteriovenous ultrasound	NA	Posttreatment drainage volume and thrombotic complication rate	Haematologic parameters, blood transfusion rate, TBL, intraoperative blood loss and bleeding complication rate

Abbreviations: BMI, body mass index; DVT, deep venous thrombosis; IQR, interquartile range; LMWH, low‐molecular‐weight heparin; SD, standard deviation; THA, total hip arthroplasty; TKA, total knee arthroplasty; VTE, venous thromboembolism.

**Table 3:** A summary of the specific VTE prophylactic aspirin/LMWH dosage used in each trial.

Study	Aspirin				LMWH				
	*n*	Dose	Frequency	Duration	Type	*n*	Dose	Frequency	Duration
Gelfer et al. [12]	61	100 mg	Daily	NA	Enoxaparin	60	40 mg	Daily	NA
Westrich et al. [41]	129	325 mg	Twice daily	4 weeks	Enoxaparin	135	30 mg as inpatient 40 mg as outpatient	Twice daily as inpatient daily as outpatient	3–5 days as inpatient 3 weeks as outpatient
Kulshrestha and Kumar [21]	194	325 mg	Twice daily	4 weeks	Enoxaparin	706	40 mg	Daily	2 weeks
Zou et al. [44]	110	100 mg	Daily	2 weeks	Enoxaparin	112	40 mg	Daily	2 weeks
CRISTAL (2022)	5416	100 mg	Daily	35 days after THA 14 days after TKA	Enoxaparin	3787	40 mg	Daily	35 days after THA 14 days after TKA
Anderson et al. [5]	380	81 mg	Daily	28 days after initial 10 days of Dalteparin	Dalteparin	398	5000 U	Daily	38 days
Zhou et al. [43]	60	100 mg	Daily	30 days	Dalteparin	60	2500 U	Daily	30 days

Abbreviations: LMWH, low‐molecular‐weight heparin; THA, total hip arthroplasty; TKA, total knee arthroplasty; VTE, venous thromboembolism.

**Table 4:** Meta‐analysis summary of primary and secondary outcomes.

Outcome	Total number aspirin, LMWH	Total events aspirin, LMWH	Heterogeneity (*I* ^2^)	*p* Value	OR (95% CI)
VTE	6350, 5258	256, 147	73%	*0.877*	0.95 (0.48–1.89)
DVT	6350, 5258	196, 123	68%	*0.965*	0.99 (0.51–1.92)
PE	5986, 4380	60, 24	0%	***0.017***	1.79 (1.11–2.89)
Major bleeding	6124, 5028	18, 22	0%	*0.424*	0.77 (0.40–1.47)
Minor bleeding	810, 1338	31, 77	3%	*0.072*	0.64 (0.40–1.04)
Mortality at 90 days	5796, 4185	4, 3	0%	*0.968*	1.03 (0.23–4.62)
*TKA subgroup*					
VTE	3841, 3370	198, 115	0%	***<0.001***	1.55 (1.21–1.98)
DVT	521, 1033	56, 57	0%	*0.315*	1.23 (0.82–1.86)
Major bleeding	323, 841	1, 6	18%	*0.886*	0.84 (0.08–9.06)
Minor bleeding	364, 878	12, 51	0%	*0.093*	0.55 (0.28–1.10)

*Note*: Bold values indicate statistically significant difference.

Abbreviations: CI, confidence interval; DVT, deep venous thrombosis; LMWH, low‐molecular‐weight heparin; OR, odds ratio; PE, pulmonary embolism; VTE, venous thromboembolism.

### Risk of VTE

**Figure 3:**
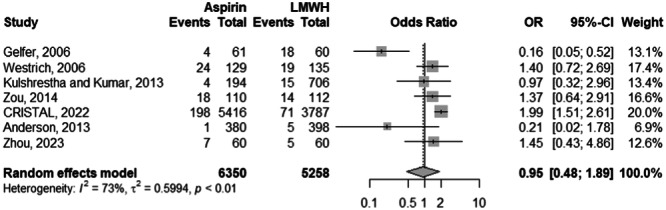
Comparison of the overall VTE (DVT and PE Combined) risk between aspirin and LMWH groups. CI, confidence interval; DVT, deep venous thrombosis; LMWH, low‐molecular‐weight heparin; OR, odds ratio; PE, pulmonary embolism; VTE, venous thromboembolism.

**Figure 4:**
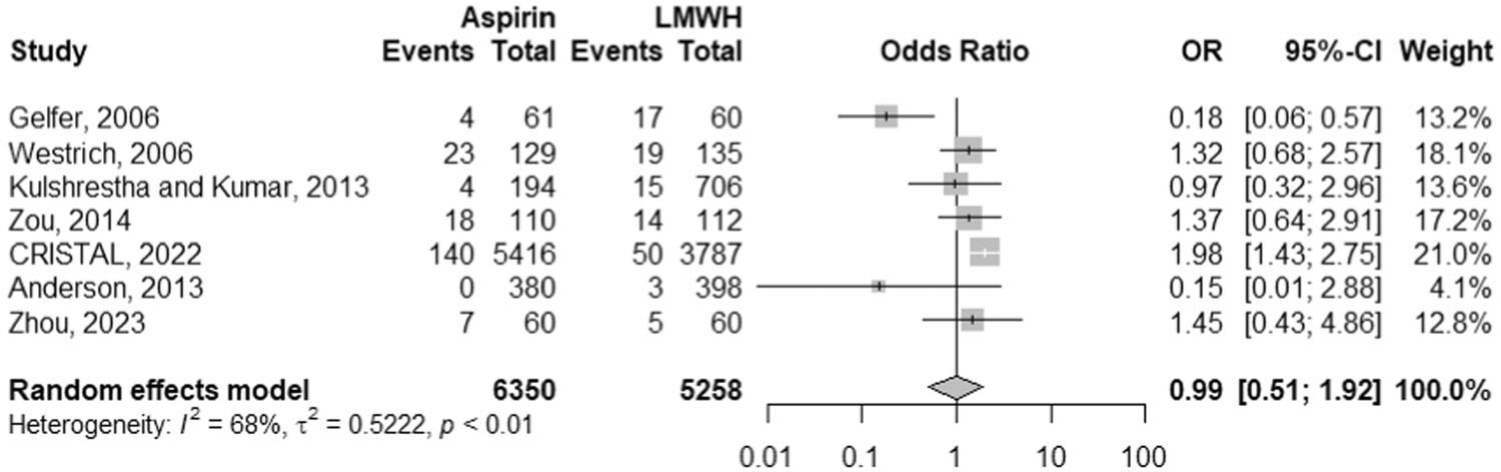
Subanalysis comparison of the DVT risk between aspirin and LMWH groups. CI, confidence interval; DVT, deep venous thrombosis; LMWH, low‐molecular‐weight heparin; OR, odds ratio.

**Figure 5:**
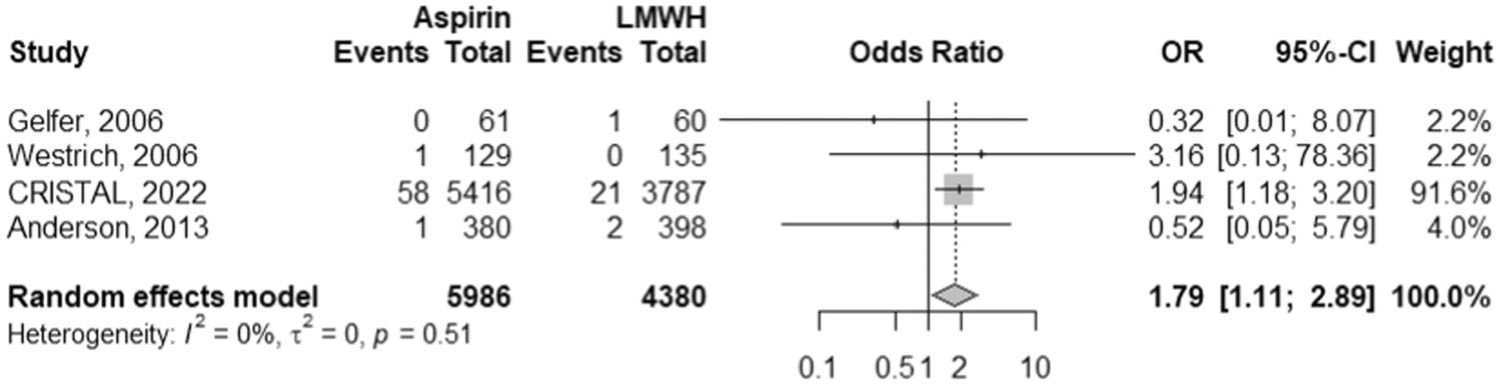
Subanalysis comparison of PE risk between aspirin and LMWH groups. CI, confidence interval; LMWH, low‐molecular‐weight heparin; OR, odds ratio; PE, pulmonary embolism.

Among 7 studies with 11,608 observations, there was no significantly different risk of VTE between the aspirin and the LMWH cohorts (odds ratio [OR]: 0.95; 95% CI: 0.48–1.89; *p*: 0.877) (Figure 3). There was significant heterogeneity for the aforementioned OR at an *I* ^2^ of 73%. When stratified by specific VTE entities (i.e., DVT or PE), the DVT risk was not significantly different between the aspirin and the LMWH cohorts (OR: 0.99; 95% CI: 0.51–1.92; *p*: 0.965) (Figure 4). Heterogeneity was significant at *I* ^2^ of 68%. However, the risk of PE, among 4 studies and 10,366 observations, was significantly higher for patients in the aspirin cohort compared to the LMWH cohort (OR: 1.79; 95% CI: 1.11–2.89; *p*: 0.017) (Figure 5). There was zero heterogeneity for the aforementioned risk statistic (*I* ^2^ = 0%).

### Risk of bleeding

**Figure 6:**
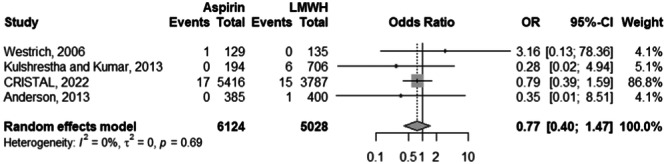
Comparison of the major bleeding risk between aspirin and LMWH groups. CI, confidence interval; LMWH, low‐molecular‐weight heparin; OR, odds ratio.

**Figure 7:**
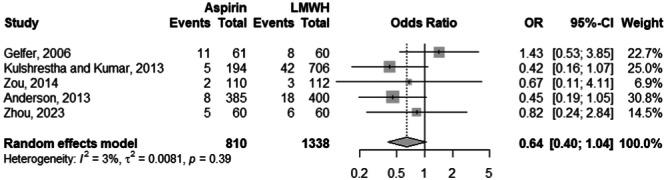
Comparison of the minor bleeding risk between aspirin and LMWH groups. CI, confidence interval; LMWH, low‐molecular‐weight heparin; OR, odds ratio.

Across 4 studies and 11,152 observations, there was no significant risk difference between the aspirin and LMWH cohorts in terms of major bleeding (OR: 0.77; 95% CI: 0.40–1.47; *p*: 0.424) (Figure 6). The aforementioned was devoid from heterogeneity at an *I* ^2^ of 0%. Similarly, the risk of minor bleeding despite being skewed towards the LMWH cohort, is not significantly different compared to the aspirin cohort (OR: 0.64; 95% CI: 0.40–1.04; *p*: 0.072) (Figure 7). An insignificant *I* ^2^ of 3% accompanies the previously mentioned risk value across 5 studies and 2148 observations.

### Risk of mortality

Due to the limited number of studies reporting mortality (*n* = 2), only the pooled risk of mortality is reported (OR: 1.03; 95% CI: 0.23–4.62; *p*: 0.968) (Supporting Information S1: Figure 1).

### Subgroup analysis

A subgroup analysis was conducted on cohorts undergoing TKA. Across 5 studies and 7259 observations, the aspirin cohort was significantly more likely to suffer VTEs compared to their LMWH counterparts (OR: 1.55; 95% CI: 1.21–1.98; *p* < 0.001) (Supporting Information S1: Figure 2). Furthermore, the risk of DVT was higher among the aspirin cohort; however, it was statistically insignificant (OR: 1.23; 95% CI: 0.82–1.86; *p*: 0.315) (Supporting Information S1: Figure 3). On the other hand, the risk of major bleeding was pooled despite the limited number of studies (OR: 0.84; 95% CI: 0.08–9.06; *p*: 0.886) (Supporting Information S1: Figure 4). Similarly, the risk of minor bleeding was insignificantly different across the two cohorts (OR: 0.55; 95% CI: 0.28–1.10; *p*: 0.093) (Supporting Information S1: Figure 5). Mortality data was not available for the TKA cohorts.

## DISCUSSION

The appropriate choice of thromboembolic prophylaxis for hip and knee arthroplasty is critically essential for operative success, both in relation to VTE prevention and managing ongoing perioperative blood loss [23, 33, 39]. We present the most up‐to‐date summation of the available literature, which can be used to make evidence‐based recommendations.

The main findings of this meta‐analysis consider both agents as effective, with a slight preference for LMWH [22]. Of note is that the incidence of PE in patients treated with aspirin appeared to be higher than that of the LMWH cohort. Proposed mechanisms for this are not overtly evident but require scrutiny, considering PE is a potentially devastating medical complication [24, 34]. The risk of bleeding appears to be clinically comparable between both cohorts, alongside the risk of mortality, reaffirming the notion that both agents present broadly similar advantageous anti‐embolism effects.

Upon subgroup analysis, LMWH appears to be more effective in reducing the rate of VTE, specifically in TKA surgery. In comparison to the broad analysis across both THA and TKR surgery, this result requires more scrutiny and may raise the question of whether specific thromboprophylaxis recommendations should be surgery‐specific and whether lower limb arthroplasty patients should not be considered one homogenous cohort [6, 20, 29].

In relation to the characteristics of the included studies, the final analysis of this paper included a significant number of patients, which is eight times more than the number of patients included in the latest Farey et al. [11] systematic review. The demographic data is comparable across both the LMWH and aspirin cohorts, which reaffirms the significance of any statistically significant conclusions drawn from the results of this analysis. The inclusion of prospective randomized trials, which represent the highest available evidence in the literature on the topic, further supports the validity of conclusions from this study. The findings from this meta‐analysis are generalizable to standard cohorts of patients undergoing lower limb arthroplasty surgery.

Critically, the rate of VTE across both cohorts was not significantly different, reaffirming the widely held notion that aspirin and LMWH are safe and effective agents in preventing VTE [22, 29, 39]. Both appear to be satisfactory low‐cost agents that can be used in patients with differing co‐morbidity profiles [7, 13, 15, 22, 39]. A recent international consensus meeting suggested that aspirin should be considered the most effective agent in preventing VTE in lower limb arthroplasty.

Several limitations have to be acknowledged within this analysis. The studies analyzed have been included over a substantial period. It is worth considering differing practices between time periods concerning more appropriate diagnostics and perioperative blood management [8, 28, 32, 37]. Also, the definition and clinical relevance of VTE require standardization in future studies, as the clinical significance of a below‐knee VTE is considered less than that of an above‐knee VTE or PE [15, 28, 39]. Given this, standardization of clinical endpoints is required in any future work, for instance, homogeneity in understanding whether the diagnosis of VTE is a negative endpoint or whether diagnosis and subsequent treatment of VTE is a negative endpoint. Heterogeneity within the available data regarding the endpoint is evident and should be addressed going forward.

## CONCLUSION

In summary, both aspirin and LMWH are safe and effective for thromboprophylaxis in lower limb arthroplasty. However, aspirin is associated with a higher risk of PE and overall VTE, particularly in knee arthroplasty, suggesting it may be less effective than LMWH. Due to heterogeneity among included studies, these findings should be interpreted cautiously, and further large‐scale randomized trials are needed.

## AUTHOR CONTRIBUTIONS

All authors contributed to the study's conception and design. Loay A. Salman, Seif B. Altahtamouni and Harman Khatkar prepared the material, reviewed the literature, collected data and assessed the quality. Abdallah Al‐Ani performed the statistical analysis. Loay A. Salman, Seif B. Altahtamouni, Harman Khatkar, Abdallah Al‐Ani, Shamsi Hameed and Abtin Alvand prepared the first draft of the manuscript, and all authors commented on previous versions of the manuscript. All authors read and approved the final manuscript.

## CONFLICT OF INTEREST STATEMENT

The authors declare no conflict of interest.

## ETHICS STATEMENT

No ethical approval is required for this study.

## Supporting information

Supporting information.

Supporting information.

## Data Availability

Data are available upon request.
