# Supplementary material for: The efficacy of aspirin versus low‐molecular‐weight heparin for venous thromboembolism prophylaxis after knee and hip arthroplasty: A systematic review and meta‐analysis of randomized controlled trials
Source: Knee Surg Sports Traumatol Arthrosc. 2024 Sep 3;33(5):1605–16. doi: 10.1002/ksa.12456 (PMC12022833; doi:10.1002/ksa.12456)
Supplement: Supplementary file 1 — Supporting information. [file KSA-33-1605-s002.docx]

**Appendix supplementary**

**Searching strategy examples:**

1)PubMed/Medline: n=226

| **Search number** | **Query** | **Search Details** | **Results** |
| --- | --- | --- | --- |
| **5** | #1 AND #2 AND #3 AND #4 | ("Aspirin"[MeSH Terms] OR "Aspirin"[All Fields] OR "aspirins"[All Fields] OR "aspirin s"[All Fields] OR "aspirine"[All Fields] OR ("Aspirin"[MeSH Terms] OR "Aspirin"[All Fields] OR ("acetylsalicylic"[All Fields] AND "acid"[All Fields]) OR "acetylsalicylic acid"[All Fields]) OR "Aspirin"[MeSH Terms]) AND ("heparin, low molecular weight"[MeSH Terms] OR ("heparin"[All Fields] AND "low molecular weight"[All Fields]) OR "low-molecular-weight heparin"[All Fields] OR ("low"[All Fields] AND "molecular"[All Fields] AND "weight"[All Fields] AND "heparin"[All Fields]) OR "low molecular weight heparin"[All Fields] OR ("heparin, low molecular weight"[MeSH Terms] OR ("heparin"[All Fields] AND "low molecular weight"[All Fields]) OR "low-molecular-weight heparin"[All Fields] OR "lmwh"[All Fields]) OR "heparin, low molecular weight"[MeSH Terms]) AND ("Venous Thromboembolism"[MeSH Terms] OR ("venous"[All Fields] AND "thromboembolism"[All Fields]) OR "Venous Thromboembolism"[All Fields] OR "VTE"[All Fields] OR ("venous thrombosis"[MeSH Terms] OR ("venous"[All Fields] AND "thrombosis"[All Fields]) OR "venous thrombosis"[All Fields] OR ("deep"[All Fields] AND "venous"[All Fields] AND "thrombosis"[All Fields]) OR "deep venous thrombosis"[All Fields]) OR "DVT"[All Fields] OR ("pulmonary embolism"[MeSH Terms] OR ("pulmonary"[All Fields] AND "embolism"[All Fields]) OR "pulmonary embolism"[All Fields]) OR ("popul econ"[Journal] OR "philos explor"[Journal] OR "pe"[All Fields]) OR "Venous Thromboembolism"[MeSH Terms]) AND ("arthroplasty, replacement, knee"[MeSH Terms] OR ("arthroplasty"[All Fields] AND "replacement"[All Fields] AND "knee"[All Fields]) OR "knee replacement arthroplasty"[All Fields] OR ("knee"[All Fields] AND "arthroplasty"[All Fields]) OR "knee arthroplasty"[All Fields] OR ("arthroplasty, replacement, knee"[MeSH Terms] OR ("arthroplasty"[All Fields] AND "replacement"[All Fields] AND "knee"[All Fields]) OR "knee replacement arthroplasty"[All Fields] OR ("knee"[All Fields] AND "replacement"[All Fields]) OR "knee replacement"[All Fields]) OR ("arthroplasty, replacement, knee"[MeSH Terms] OR ("arthroplasty"[All Fields] AND "replacement"[All Fields] AND "knee"[All Fields]) OR "knee replacement arthroplasty"[All Fields] OR ("total"[All Fields] AND "knee"[All Fields] AND "arthroplasty"[All Fields]) OR "total knee arthroplasty"[All Fields]) OR ("arthroplasty, replacement, knee"[MeSH Terms] OR ("arthroplasty"[All Fields] AND "replacement"[All Fields] AND "knee"[All Fields]) OR "knee replacement arthroplasty"[All Fields] OR ("total"[All Fields] AND "knee"[All Fields] AND "replacement"[All Fields]) OR "total knee replacement"[All Fields]) OR "TKA"[All Fields] OR (("hip"[MeSH Terms] OR "hip"[All Fields]) AND ("arthroplasty"[MeSH Terms] OR "arthroplasty"[All Fields] OR "arthroplasties"[All Fields])) OR ("arthroplasty, replacement, hip"[MeSH Terms] OR ("arthroplasty"[All Fields] AND "replacement"[All Fields] AND "hip"[All Fields]) OR "hip replacement arthroplasty"[All Fields] OR ("hip"[All Fields] AND "replacement"[All Fields]) OR "hip replacement"[All Fields]) OR ("arthroplasty, replacement, hip"[MeSH Terms] OR ("arthroplasty"[All Fields] AND "replacement"[All Fields] AND "hip"[All Fields]) OR "hip replacement arthroplasty"[All Fields] OR ("total"[All Fields] AND "hip"[All Fields] AND "arthroplasty"[All Fields]) OR "total hip arthroplasty"[All Fields]) OR ("arthroplasty, replacement, hip"[MeSH Terms] OR ("arthroplasty"[All Fields] AND "replacement"[All Fields] AND "hip"[All Fields]) OR "hip replacement arthroplasty"[All Fields] OR ("total"[All Fields] AND "hip"[All Fields] AND "replacement"[All Fields]) OR "total hip replacement"[All Fields]) OR "THA"[All Fields] OR "arthroplasty, replacement, knee"[MeSH Terms] OR "arthroplasty, replacement, hip"[MeSH Terms]) | 226 |
| **4** | knee arthroplasty OR knee replacement OR total knee arthroplasty OR total knee replacement OR TKA OR hip arthroplasty OR hip replacement OR total hip arthroplasty OR total hip replacement OR THA OR ("Arthroplasty, Replacement, Knee"[Mesh]) OR "Arthroplasty, Replacement, Hip"[Mesh] | "arthroplasty, replacement, knee"[MeSH Terms] OR ("arthroplasty"[All Fields] AND "replacement"[All Fields] AND "knee"[All Fields]) OR "knee replacement arthroplasty"[All Fields] OR ("knee"[All Fields] AND "arthroplasty"[All Fields]) OR "knee arthroplasty"[All Fields] OR ("arthroplasty, replacement, knee"[MeSH Terms] OR ("arthroplasty"[All Fields] AND "replacement"[All Fields] AND "knee"[All Fields]) OR "knee replacement arthroplasty"[All Fields] OR ("knee"[All Fields] AND "replacement"[All Fields]) OR "knee replacement"[All Fields]) OR ("arthroplasty, replacement, knee"[MeSH Terms] OR ("arthroplasty"[All Fields] AND "replacement"[All Fields] AND "knee"[All Fields]) OR "knee replacement arthroplasty"[All Fields] OR ("total"[All Fields] AND "knee"[All Fields] AND "arthroplasty"[All Fields]) OR "total knee arthroplasty"[All Fields]) OR ("arthroplasty, replacement, knee"[MeSH Terms] OR ("arthroplasty"[All Fields] AND "replacement"[All Fields] AND "knee"[All Fields]) OR "knee replacement arthroplasty"[All Fields] OR ("total"[All Fields] AND "knee"[All Fields] AND "replacement"[All Fields]) OR "total knee replacement"[All Fields]) OR "TKA"[All Fields] OR (("hip"[MeSH Terms] OR "hip"[All Fields]) AND ("arthroplasty"[MeSH Terms] OR "arthroplasty"[All Fields] OR "arthroplasties"[All Fields])) OR ("arthroplasty, replacement, hip"[MeSH Terms] OR ("arthroplasty"[All Fields] AND "replacement"[All Fields] AND "hip"[All Fields]) OR "hip replacement arthroplasty"[All Fields] OR ("hip"[All Fields] AND "replacement"[All Fields]) OR "hip replacement"[All Fields]) OR ("arthroplasty, replacement, hip"[MeSH Terms] OR ("arthroplasty"[All Fields] AND "replacement"[All Fields] AND "hip"[All Fields]) OR "hip replacement arthroplasty"[All Fields] OR ("total"[All Fields] AND "hip"[All Fields] AND "arthroplasty"[All Fields]) OR "total hip arthroplasty"[All Fields]) OR ("arthroplasty, replacement, hip"[MeSH Terms] OR ("arthroplasty"[All Fields] AND "replacement"[All Fields] AND "hip"[All Fields]) OR "hip replacement arthroplasty"[All Fields] OR ("total"[All Fields] AND "hip"[All Fields] AND "replacement"[All Fields]) OR "total hip replacement"[All Fields]) OR "THA"[All Fields] OR "arthroplasty, replacement, knee"[MeSH Terms] OR "arthroplasty, replacement, hip"[MeSH Terms] | 110,547 |
| **3** | venous thromboembolism OR VTE OR deep venous thrombosis OR DVT OR pulmonary embolism OR PE OR "Venous Thromboembolism"[Mesh] | "Venous Thromboembolism"[MeSH Terms] OR ("venous"[All Fields] AND "thromboembolism"[All Fields]) OR "Venous Thromboembolism"[All Fields] OR "VTE"[All Fields] OR ("venous thrombosis"[MeSH Terms] OR ("venous"[All Fields] AND "thrombosis"[All Fields]) OR "venous thrombosis"[All Fields] OR ("deep"[All Fields] AND "venous"[All Fields] AND "thrombosis"[All Fields]) OR "deep venous thrombosis"[All Fields]) OR "DVT"[All Fields] OR ("pulmonary embolism"[MeSH Terms] OR ("pulmonary"[All Fields] AND "embolism"[All Fields]) OR "pulmonary embolism"[All Fields]) OR ("popul econ"[Journal] OR "philos explor"[Journal] OR "pe"[All Fields]) OR "Venous Thromboembolism"[MeSH Terms] | 237,713 |
| **2** | low-molecular-weight heparin OR LMWH OR "Heparin, Low-Molecular-Weight"[Mesh] | "heparin, low molecular weight"[MeSH Terms] OR ("heparin"[All Fields] AND "low molecular weight"[All Fields]) OR "low-molecular-weight heparin"[All Fields] OR ("low"[All Fields] AND "molecular"[All Fields] AND "weight"[All Fields] AND "heparin"[All Fields]) OR "low molecular weight heparin"[All Fields] OR ("heparin, low molecular weight"[MeSH Terms] OR ("heparin"[All Fields] AND "low molecular weight"[All Fields]) OR "low-molecular-weight heparin"[All Fields] OR "lmwh"[All Fields]) OR "heparin, low molecular weight"[MeSH Terms] | 22,221 |
| **1** | Aspirin OR acetylsalicylic acid OR "Aspirin"[Mesh] | "Aspirin"[MeSH Terms] OR "Aspirin"[All Fields] OR "aspirins"[All Fields] OR "aspirin s"[All Fields] OR "aspirine"[All Fields] OR ("Aspirin"[MeSH Terms] OR "Aspirin"[All Fields] OR ("acetylsalicylic"[All Fields] AND "acid"[All Fields]) OR "acetylsalicylic acid"[All Fields]) OR "Aspirin"[MeSH Terms] | 79,322 |

2)Embase: n= 585

('acetylsalicylic acid'/exp OR 'acetylsalicylic acid') AND ('low molecular weight heparin'/exp OR 'low molecular weight heparin') AND ('venous thromboembolism'/exp OR 'venous thromboembolism' OR 'deep vein thrombosis'/exp OR 'deep vein thrombosis' OR 'lung embolism'/exp OR 'lung embolism') AND ('total knee arthroplasty'/exp OR 'total knee arthroplasty' OR 'total hip replacement'/exp OR 'total hip replacement')

3)Cochrane Library: n=13

ID Search Hits

#1 MeSH descriptor: [Arthroplasty] explode all trees 8043

#2 MeSH descriptor: [Venous Thromboembolism] explode all trees 1326

#3 MeSH descriptor: [Aspirin] explode all trees 7981

#4 MeSH descriptor: [Heparin, Low-Molecular-Weight] explode all trees 2740

#5 #1 AND #2 AND #3 AND #4 13

**“PICOs” framework adopted:**

| **Population** | Inclusion: Adult patients > 18 years old, undergoing primary TKA or THA indicated primarily for OA. Regardless of gender, BMI and ethnicity.  Exclusion: patients under 18, undergoing revision surgery, or due to secondary osteoarthritis. |
| --- | --- |
| **Intervention** | Aspirin only as the primary thrombophylactic agent |
| **Comparators** | LMWH only as the primary thrombophylactic agent |
| **Outcomes** | **Primary Outcome:** VTE (PE, DVT, or both)  **Secondary outcomes:**  -Minor bleeding  -Major bleeding  -Mortality within 90 days after surgery |

**sFigure 1.** Comparison of the mortality rate risk between Aspirin and LMWH groups. OR: Odds ratio, CI: confidence interval.


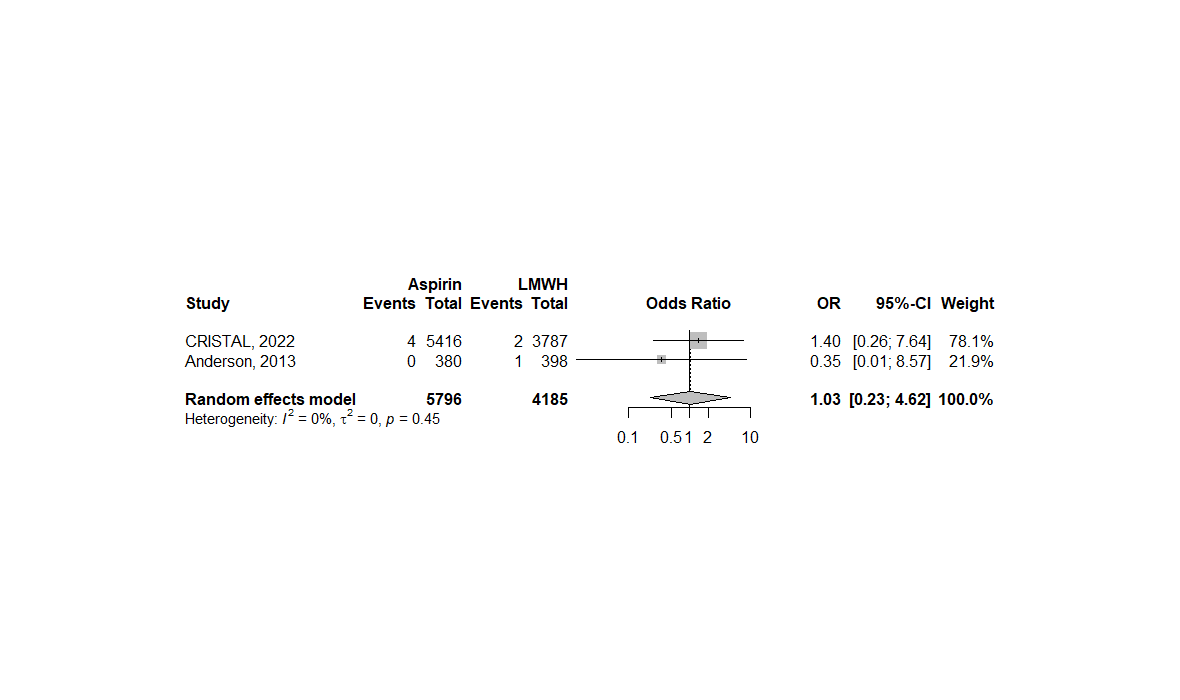


**sFigure 2.** Comparison of the VTE risk between Aspirin and LMWH groups in TKA. OR: Odds ratio, CI: confidence interval.


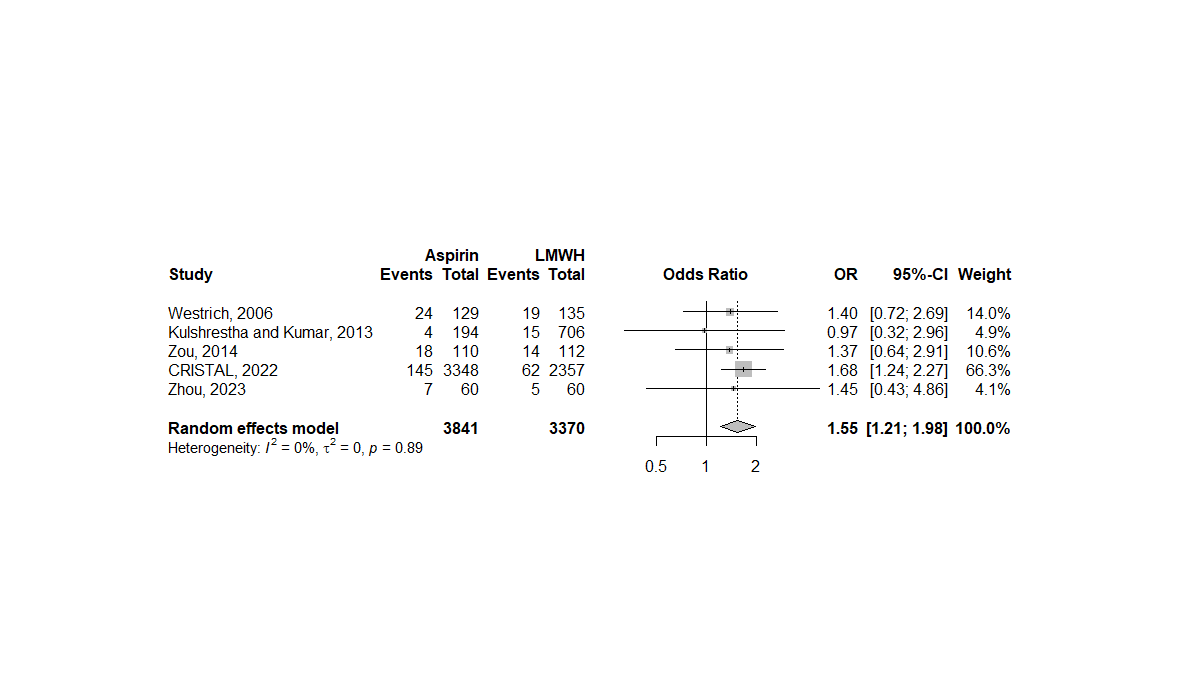


**sFigure 3.** Comparison of the DVT risk between Aspirin and LMWH groups in TKA. OR: Odds ratio, CI: confidence interval.


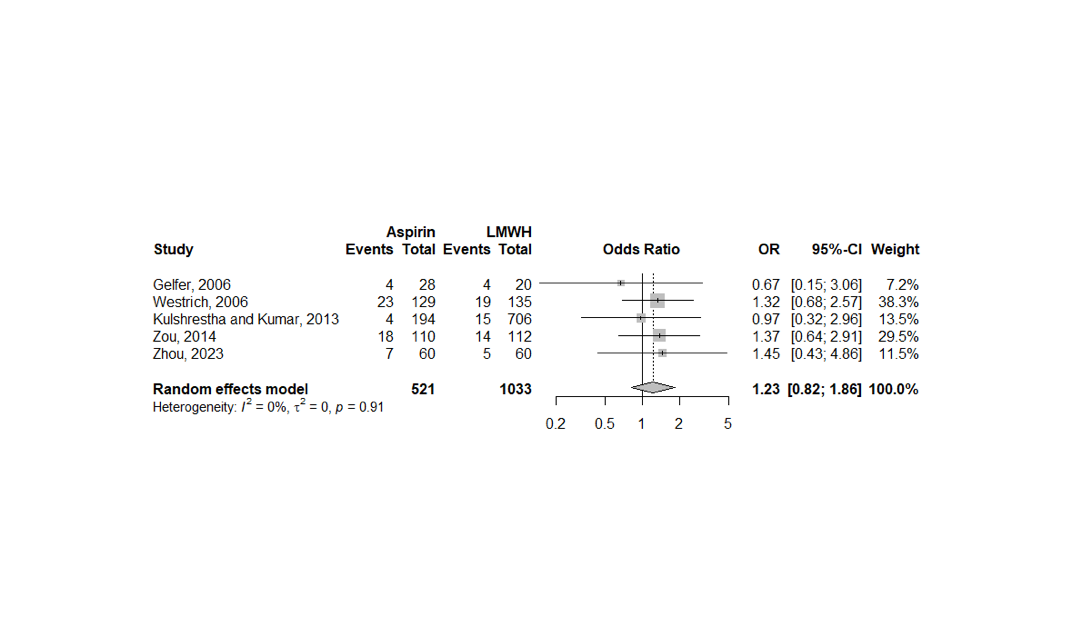


**sFigure 4.** Comparison of the major bleeding risk between Aspirin and LMWH groups in TKA. OR: Odds ratio, CI: confidence interval

**
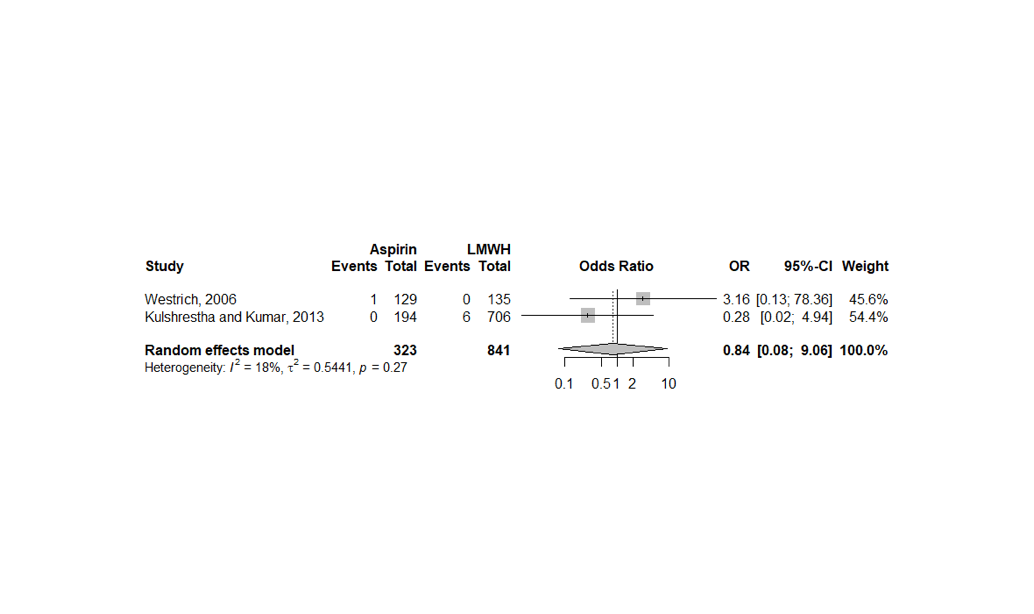
**

**sFigure 5.** Comparison of the minor bleeding risk between Aspirin and LMWH groups in TKA. OR: Odds ratio, CI: confidence interval


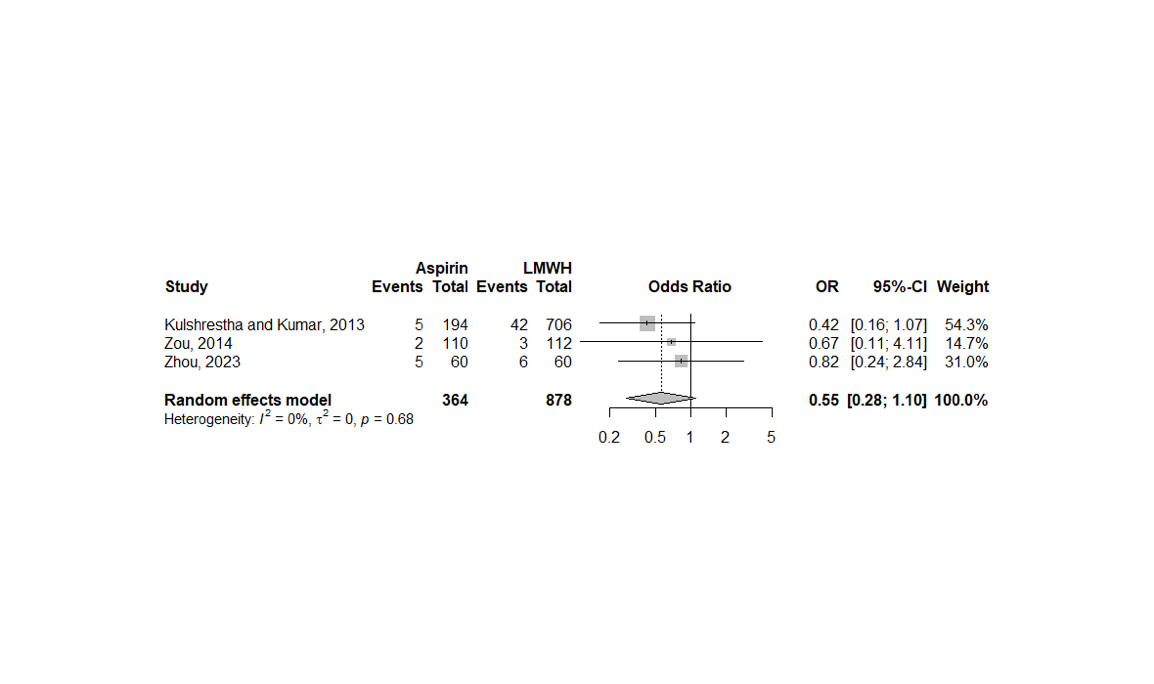


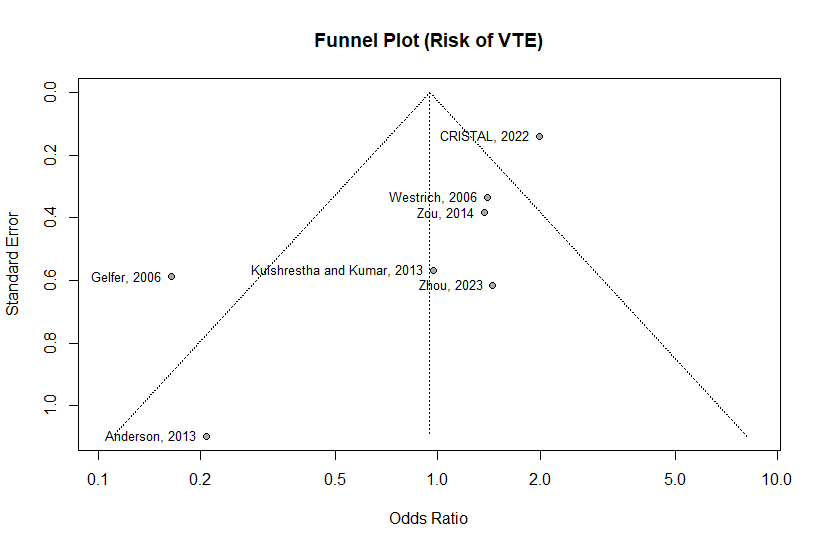

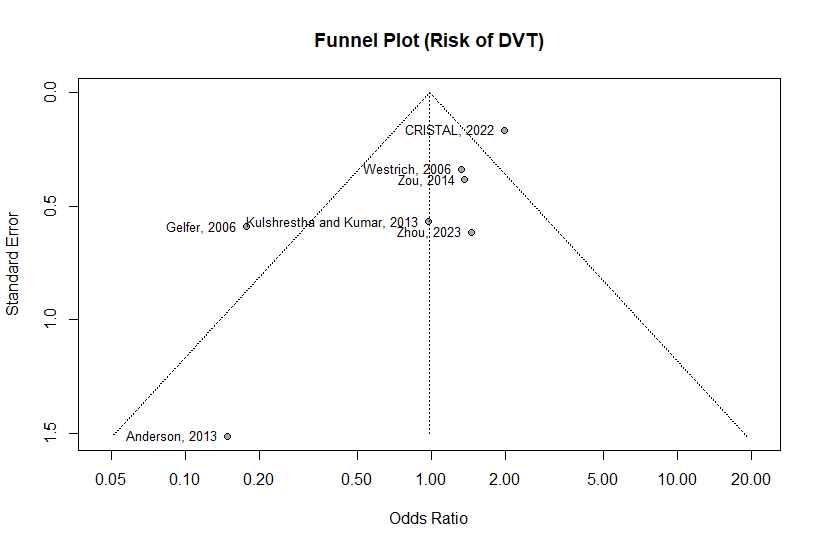

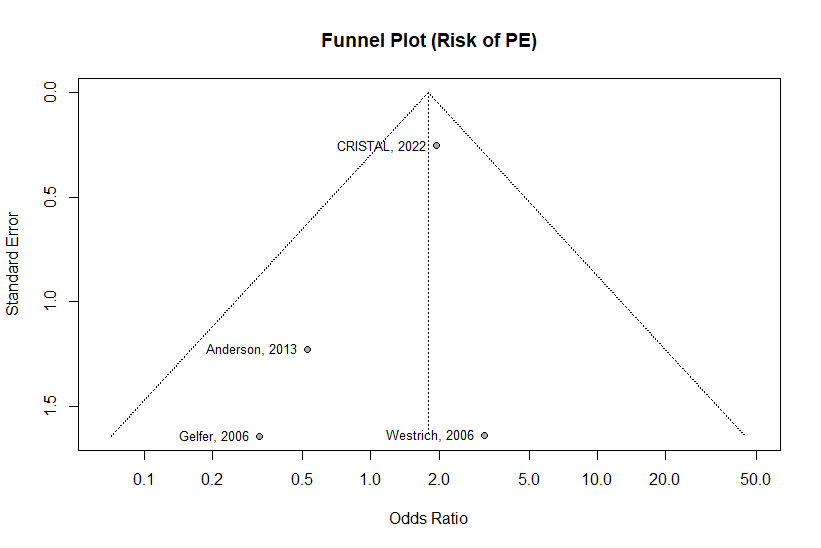

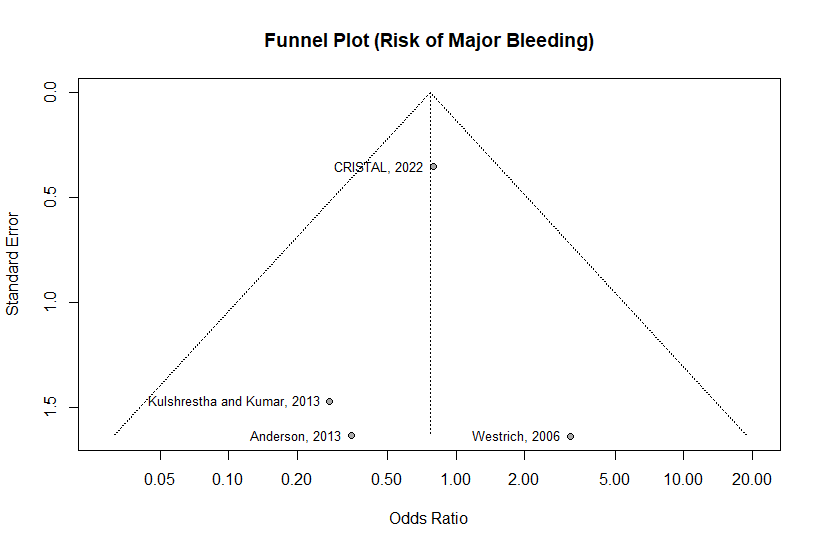

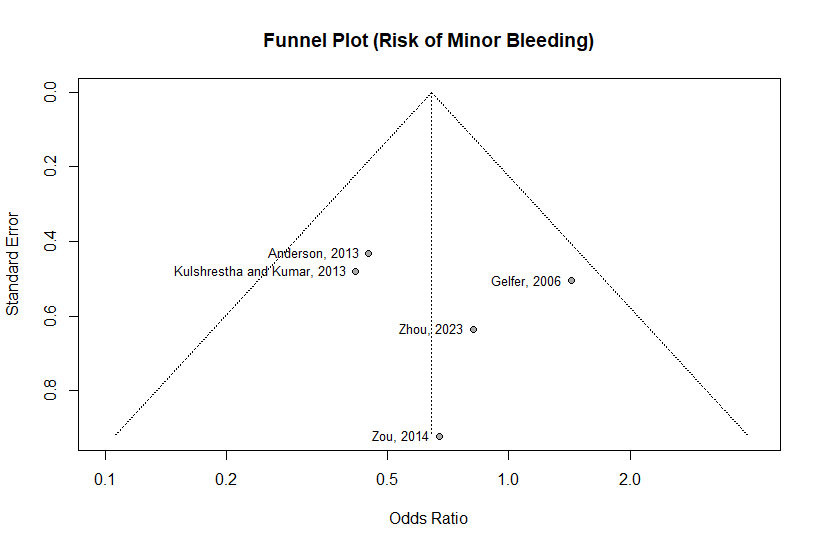

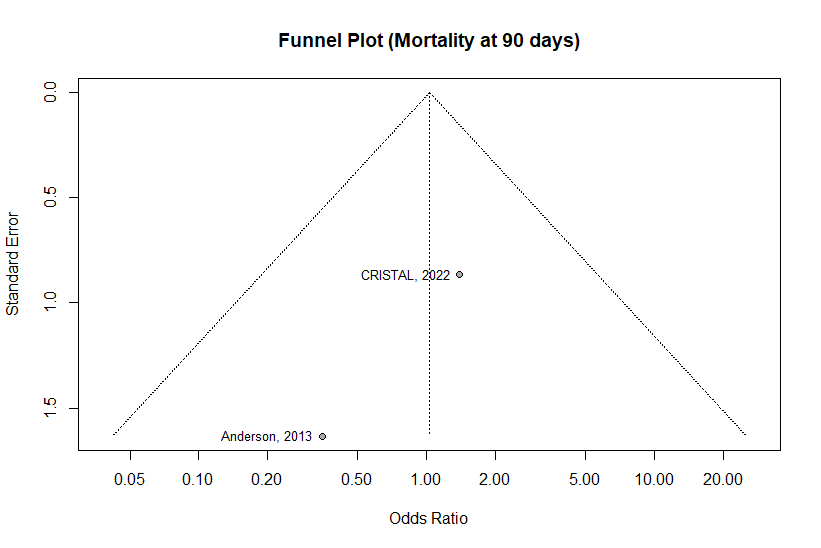


**Factors affecting quality of evidence using GRADEpro tool**

| Outcomes | Study Design | Risk of bias | Inconsistency of results | Indirectness of evidence | Imprecision | Publication bias |
| --- | --- | --- | --- | --- | --- | --- |
| Gelfer, 2006 | ⨁⨁⨁⨁ | ⨁⨁⨁⨁ | ⨁⨁⨁◯ | ⨁⨁⨁⨁ | ⨁⨁⨁◯ | Refer to Funnel Plots |
| Westrich, 2006 | ⨁⨁⨁⨁ | ⨁⨁⨁◯ | ⨁⨁⨁⨁ | ⨁⨁⨁⨁ | ⨁◯◯◯ | Refer to Funnel Plots |
| Kulshrestha and Kumar, 2013 | ⨁⨁⨁⨁ | ⨁⨁⨁⨁ | ⨁⨁⨁◯ | ⨁⨁⨁⨁ | ⨁◯◯◯ | Refer to Funnel Plots |
| Zou, 2014 | ⨁⨁⨁⨁ | ⨁⨁⨁◯ | ⨁⨁⨁⨁ | ⨁⨁⨁⨁ | ⨁◯◯◯ | Refer to Funnel Plots |
| CRISTAL, 2022 | ⨁⨁⨁⨁ | ⨁⨁⨁⨁ | ⨁⨁⨁⨁ | ⨁⨁⨁⨁ | ⨁⨁⨁◯ | Refer to Funnel Plots |
| Anderson, 2013 | ⨁⨁⨁⨁ | ⨁⨁⨁⨁ | ⨁⨁◯◯ | ⨁⨁⨁⨁ | ⨁◯◯◯ | Refer to Funnel Plots |
| Zhou, 2023 | ⨁⨁⨁⨁ | ⨁⨁⨁⨁ | ⨁⨁⨁◯ | ⨁⨁⨁⨁ | ⨁◯◯◯ | Refer to Funnel Plots |

**JADAD Score summary of the included trials**

| Outcomes | Randomization | Appropriate Randomization | Blinding | Appropriate Blinding | Follow-up/dropouts |  | Overall |
| --- | --- | --- | --- | --- | --- | --- | --- |
| Gelfer, 2006 | 1 | 0 | 1 | 1 | 1 |  | 3 |
| Westrich, 2006 | 0 | 0 | 1 | 1 | 1 |  | 3 |
| Kulshrestha and Kumar, 2013 | 1 | 1 | 1 | 1 | 1 |  | 5 |
| Zou, 2014 | 1 | 0 | 1 | 1 | 1 |  | 3 |
| CRISTAL, 2022 | 1 | 0 | 1 | 1 | 1 |  | 3 |
| Anderson, 2013 | 1 | 1 | 1 | 1 | 1 |  | 5 |
| Zhou, 2023 | 1 | 1 | 1 | 1 | 1 |  | 5 |
